# Speedy Routing Recovery Protocol for Large Failure Tolerance in Wireless Sensor Networks

**DOI:** 10.3390/s100403389

**Published:** 2010-04-07

**Authors:** Joa-Hyoung Lee, In-Bum Jung

**Affiliations:** Department of Computer Science and Engineering, Kangwon National University, Chuncheon Gangwondo, 200-701, Korea; E-Mail: jinnie4u@kangwon.ac.kr

**Keywords:** failure tolerance, routing interval, failure detection, failure recovery

## Abstract

Wireless sensor networks are expected to play an increasingly important role in data collection in hazardous areas. However, the physical fragility of a sensor node makes reliable routing in hazardous areas a challenging problem. Because several sensor nodes in a hazardous area could be damaged simultaneously, the network should be able to recover routing after node failures over large areas. Many routing protocols take single-node failure recovery into account, but it is difficult for these protocols to recover the routing after large-scale failures. In this paper, we propose a routing protocol, referred to as ARF (***A***daptive routing protocol for fast ***R***ecovery from large-scale ***F***ailure), to recover a network quickly after failures over large areas. ARF detects failures by counting the packet losses from parent nodes, and upon failure detection, it decreases the routing interval to notify the neighbor nodes of the failure. Our experimental results indicate that ARF could provide recovery from large-area failures quickly with less packets and energy consumption than previous protocols.

## 1. Introduction

Recent advances in MEMS (Micro Electro Mechanical Systems) and microprocessor and wireless communication technologies have enabled the deployment of large-scale sensor networks, where thousands or even tens of thousands of small sensors are distributed over a vast field to obtain sensing data. Sensor networks have attracted significant attention as important infrastructure for data collection in an environment for pervasive computing. In this field, wireless sensor networks play a special role in home automation, environmental monitoring, military, health, and other applications.

In particular, wireless sensor networks have important applications in hazardous areas such as battle fields or disaster areas, where access by humans is difficult. However, in addition to being dangerous for humans, the hazards in these areas might also threaten sensor nodes, which are often physically fragile. Usually, sensor nodes are designed to be cheap, very small, and use very limited resources. Therefore, they should not be regarded as strong enough to resist a physical impact. This fragile characteristic of sensor nodes makes a wireless sensor network vulnerable to the hazards in such areas. If some of the sensor nodes in a wireless sensor network deployed in a hazardous area become damaged and can no longer work properly, the data from the area covered by these damaged sensor nodes cannot be collected properly. Therefore, these sensor nodes should be repaired or covered by other sensor nodes.

**Figure 1.**
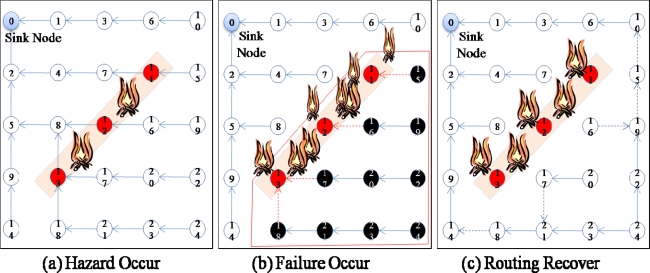
Node failure and routing recovery.

Moreover, the damaged sensor nodes could also obstruct the sink node from collecting data from behind the area of the damaged sensor nodes because these sensor nodes are not only data sources but also routing (relaying) nodes for other source nodes. For example, in Figure 1, when a hazard (fire) occurs in the sensor network (Figure 1a), some sensor nodes (sensor nodes 11–13) fail, and thus, these nodes are no longer able to forward data from other sensor nodes (sensor nodes 15–24) in the lower part of the routing tree (Figure 1b). In this case, although only 3 sensor nodes (sensor nodes 11–13) fail and the child nodes (sensor nodes 15–24) of these sensor nodes are still working properly, packets from the child nodes (sensor nodes 15–24) are not able to arrive at the sink node because the parent nodes (sensor nodes 11–13) have failed. Data from the child nodes (sensor nodes 15–24) should be delivered to the sink nodes, and therefore, the routing paths should be reestablished using the available nodes connected to a sink node to recover from the failure (Figure 1c). Hence, for reliable data transfer, routing protocols should be aware of failures and have the ability to recover from them quickly.

Node failures in a large area can be regarded as a routing hall in the geographic routing protocol which works based on the geographic coordinates and thus be recovered fast since the geographic routing protocol usually has the ability to detour the routing hall. However, the geographic routing requires that sensor nodes have to know their location on the network field. Localization of a sensor node is in need of a special device to estimate the location such as GPS or Ultra Sound Emitter which is expensive or has the limited usage. On the other hand, the tree based routing protocols doesn’t require any special device and thus can be used in any case without additional cost. As a consequence, the tree based routing protocol is still used in many area and the node failures in tree based routing can be a big problem. Therefore, we concentrate to the node failures in large area with tree based routing protocol.

Usually, routing protocols for wireless sensor networks have the ability to recover from the failure of a sensor node. In these protocols, if a sensor node cannot receive messages from surrounding nodes for a specified period of time, the sensor node is classified as a failed node and thus excluded from the routing path. In the case of the failure of a single node, this mechanism works properly without much overhead. However, if more than two sensor nodes fail in the same area, as shown in Figure 1, this mechanism might not work because the other nodes might not find a path to the sink. Therefore, the rapid recovery from large-scale failures should be considered when designing routing protocols.

Fast recovery from large failures has mainly been studied for geographic routing protocols. In geographic routing protocols, packets are forwarded based on geographic coordinates, and therefore, node failures should not be a big problem because sensor nodes do not maintain a routing table, but just forward packets based on the coordinates and available neighbor nodes. Large-scale node failures might affect the routing process momentarily, but packets would be forwarded through normal sensor nodes toward a sink node. However, tree-based routing protocols usually only consider single node failure recovery methods, which are not scalable for large-scale failures. Tree-based routing could be much more affected by large-scale failures because sensor nodes choose the next node to forward a packet based on cumulated information. Moreover, in tree-based routing, sensor nodes do not have a view of the whole network. Therefore, sensor nodes that are confused due to failures might not be able to quickly find a new path to the correct nodes. Thus, fast recovery from large failures should also be considered in tree-based routing.

Failure recovery in tree-based routing mainly depends on the transmission intervals for routing control messages, which include information on the node and link states. The time needed to recover from the failed routing should be proportional to the interval for routing control messages. Sensor nodes should exchange routing control messages quickly in the case of failure. However, because transmitting routing control messages requires large energy consumptions by sending and receiving nodes, most routing protocols set up a long transmission interval for routing control messages to reduce the energy consumption. As a consequence, it takes a long time for a network to recover when a routing tree is collapsed by node failures in conventional tree-based routing methods. To quickly recover the collapsed routing tree in an energy-efficient manner, the transmission interval for the routing control messages should be sufficiently long to reduce the energy consumption under normal conditions, while routing control messages should be exchanged quickly enough to recover from a failed routing path when several nodes fail concurrently.

This paper proposes a routing protocol called ARF (***A***daptive routing protocol for fast ***R***ecovery from large-scale ***F***ailure). This protocol is energy efficient, yet provides rapid recovery from a failed routing tree covering a large area. ARF detects a node failure by counting the packet losses from neighbor nodes. If a sensor node has not listened to its neighboring sensor nodes for a specified period, that sensor node is regarded by its neighbor sensors as a failed node and is removed from the neighbor table. In a case where the parent node of a sensor node has failed, ARF tries to find a new parent in the neighbor table. When there is no candidate for a parent in the neighbor tree, ARF labels the sensor node as an orphan and shortens the transmission interval for routing control messages to notify the neighbor nodes that it is an orphan. If a sensor node receives a routing control message with an orphan notification and it has a reliable parent, then the sensor node also shortens its routing transmission interval to aid the orphan node. If an orphan node receives a routing control message from a proper parent node, then the orphan node regards the routing control message source node as its new parent node and returns to the long transmission interval. Our experimental results indicated clearly that ARF could recover from large-area failures quickly with less packets than previous protocols. In the next section, we will review related studies. Section 3 describes ARF, and Section 4 provides a performance evaluation. We conclude the paper in Section 5.

## 2. Related Studies

It is important to note the differences between faults, errors, and failures. A fault is any kind of defect that leads to an error. An error corresponds to an incorrect system state. Such a state may lead to a failure. A failure is the manifestation of an error, which occurs when the system deviates from its specification and cannot deliver its intended functionality. A sensor node with a fault or error could transmit a packet that might have incorrect data or in an incorrect way. Some researches focus on the detection and exclusion of faulty or erroneous nodes. If several sensor nodes in one area acquire routing layer faults, packets might be transferred in the wrong direction. Such packets could later be delivered by normal sensor nodes. However, if several nodes in an area fail simultaneously, packets from the failed area could be lost because the sensor nodes in the failed area could not forward them. Therefore, failures could cause more serious problems in wireless sensor networks [1–18].

ARRIVE is a probabilistic algorithm that leverages the high node density and the inherent broadcast medium found in sensor networks to achieve routing robust to both link failures and patterned node failures without resorting to periodic flooding of the network [9]. ARRIVE is based on a tree-like topology rooted at the sink of the network, and nodes use localized observed behavior of the surrounding nodes to make probabilistic decisions for forwarding packets. ARRIVE adapts to large patterned failures within a relatively short period of time at the cost of only moderate increases in overall power consumption and source-to-sink latency. Shortest Path Minded SPIN (SPMS) in which every node has a zone defined by its maximum transmission radius was proposed in [10]. A node which is a data source advertises the availability of data to all the nodes in its zone using a metadata descriptor. Any interested node requests the data and gets sent the data using multi-hop communication via the shortest path. The failure of any node in the path is detected and recovered using backup route.

Conventional routing protocols such as MintRoute or PROC (Proactive Routing with Coordination) use the cumulative statistics for the links between nodes to detect failures. If a sensor node cannot receive packets from a neighbor node, the neighbor node should be regarded as a failed node. If a neighbor node is classified as a failed node, the node is removed from the neighbor table. A sensor node updates its neighbor table whenever it receives a message from a neighbor node. If the packet is a routing packet, the packet includes the state information of the sending node. The routing control message tells the receiving node how good the sending node is. When a data packet is received, the receiving node updates the link statistic for the sending node. A sensor node checks its neighbor table frequently and selects a neighbor node with the highest link quality as a parent node. If a sensor node has not heard from a neighbor node for a while, then that neighbor node is regarded as a failed node and removed from the neighbor table. Some researchers have proposed using multiple paths to send a packet for reliability. In these protocols, a packet is sent through multiple paths that are far away from each other, and thus, multiple copies of the packet could arrive at a sink node, even if some paths fail. However, although packets could be delivered to a sink node properly, multiple transmissions of a packet require more energy consumption. Thus, the lifetime of a sensor network could be shortened quickly [19–25].

## 3. ARF

### 3.1. Architecture

We consider a densely deployed wireless sensor network such that each sensor node has one or more sensing devices. A sensor node frequently transfers the sensed data to a sink node or gateway. Sensor nodes should control the data transmission interval in an inverse proportion to the variation in a phenomenon. A tree-based routing protocol is used to construct the path from sensor nodes to the sink node. The path to the sink node can be changed whenever the link states to the upper nodes are unreliable due to node failure or obstacles. The lower nodes in a routing tree have to compete with other nodes for an upper node in a routing tree. We assume that sensor nodes are always awake, however a sensor node can go to sleep frequently if the transition frequency is known to others with presetting or routing control message.

The architecture and operation of ARF is similar to other tree-based routing methods, except for its routing recovery, which is designed to allow the routing tree to recover from large-scale failures. ARF consists of a Routing Table, Table Manager, Link Estimator, Parent Selector, Cycle Detector, Forwarding Module, Routing Recoverer, Timer, and Dispatcher, as listed in Table 1.

Routing Table maintains a list of neighbor sensor nodes, routing information, and link quality. The Routing Table in ARF consists of the Node ID, Hop Count, Parent ID, Transfer Packets, Received Packets, Packet Sequence, Lost Packets, Link Quality, and Orphan Flag, as listed in Table 2. The Hop Count is used to find the shortest path to a sink node. A neighbor sensor node with the smallest Hop Count becomes a parent node. The Parent ID is used to detect and prevent a loop or cycle. Transfer Packets is the number of packets sent to the neighbor node successfully. Received Packets is the number of packets received from the neighbor node, which are either data messages or routing control messages. The Transferred Packets and Received Packets are used by the Link Estimator to compute the Link Quality. The Packet Sequence is updated with the sequence field of a data message and used to detect a packet loss. Whenever a packet loss is detected by comparing the sequence numbers, the number of Lost Packets increases. Orphan Flag is used to represent the Orphan state. If a sensor node does not have a parent node, the sensor node is classified as an orphan node. The Routing Table is used when the Parent Selector chooses a parent.

The Routing Table is updated by the Table Manager and Link Estimator whenever a message is received. The received message could be a data message or a routing control message. If it is a data message, the Received Packets and Packet Sequence of the Routing Table entry that contains information about the node that sent the received message are updated by the Table Manager. In addition, if the difference in the packet sequences of the current packet and last packet is more than 1, Lost Packets is updated. If it is a routing control message, it contains state information about the sending node; thus, the Table Manager not only updates the message receive count but also other attributes such as the Hop count and Parent ID. If a sensor node has not received any packet from a neighbor node, the Table Manager classifies the neighbor node as a failed node and evicts the neighbor node from the Routing Table.

The Link Estimator computes the link qualities to neighbor nodes based on the Transferred Packets, Received Packets, and Lost Packets. The Cycle Detector detects a loop or cycle. When a packet sent by the sensor node itself is received by the sensor node again, it is regarded as a loop or cycle and the packet is dropped. The Forwarding Module transfers data packets from its own higher applications or from a child node toward the parent node. For reliable transmission, the Sending Queue is used. The Timer is used to set the intervals for routing control messages, link estimation, and parent selection. The Timer interval can be changed by the Routing Recoverer. The Parent Selector chooses a parent node with the smallest hop count and highest link quality. If there is no proper node for a parent node, the Parent Selector sets the node as an Orphan and starts the Routing Recoverer. The Routing Recoverer sets up a short routing interval for the rapid spread of the orphan notification by changing the Timer period.

**Figure 2.**
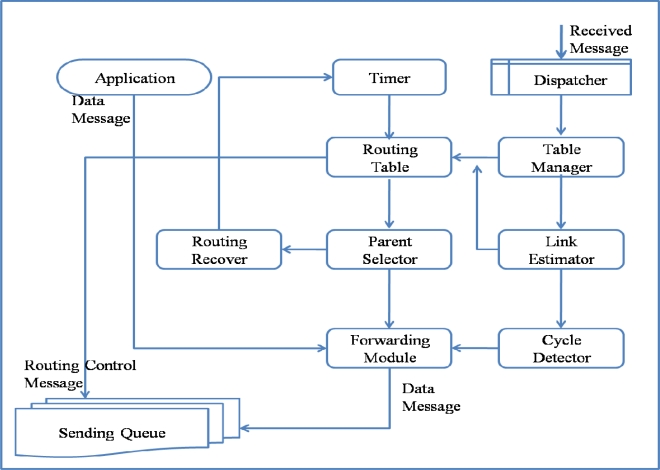
Operation of ARF.

Figure 2 shows the operation of ARF. A packet could be a data message generated by the application layer of the node itself, a routing control message generated by the Routing Table, or a message received from a neighbor node. A data packet from the application layer is directly input to the Forwarding Module, which simply sets the parent ID field using the parent ID in the Routing Table and puts it into the Sending Queue. The Routing Table generates a routing control message at the transmission interval for routing control messages, as controlled by the Timer. A routing control message contains information extracted from the Routing Table. A routing control message is inserted directly into the Sending Queue with a broadcast address and thus could be received by neighbor nodes. A received message is dispatched by the Dispatcher from the Receive Queue and is either a routing control message or a data message. All of the messages transmitted around a sensor node should be received by overhearing for link estimation and failure detection. Both received routing and data messages are used by the Table Manager to update the Routing Table. A received data message that has passed through the Cycle Detector filter is input to the Forwarding Module and Sending Queue.

### 3.2. Routing Recovery

For fast routing recovery, the Parent Selector and Routing Recoverer of ARF work differently from conventional routing protocols. In a conventional routing protocol, the Parent Selector selects the sensor node with the lowest hop count and highest link quality. ARF does something similar. However, in a conventional routing protocol, if a sensor node does not receive packets from the parent node for a specified period, the parent node is removed from the Routing Table and a new parent node is selected from the remaining neighbor nodes. If all of the nodes at the upper level of the routing tree fail, conventional routing protocols choose a node at same level as the sensor node itself, or even at a lower level. If packet transmission toward all of the neighbor nodes is unsuccessful, routing is regarded as failed. As a result, it takes a long time to detect routing failures in conventional routing protocols.

On the other hand, in ARF, the Parent Selector chooses a parent only from the group of nodes with the lowest hop count. Because we assume a densely deployed sensor network, a sensor node could have several nodes with the same hop count. If all of the nodes with the lowest hop count have failed, the Parent Selector in ARF regards itself as an orphan node without a parent node and sets its parent node id and hop count to predefined maximum values. With the modified Parent Selector, ARF can detect a failure at the upper level of a routing tree faster than conventional routing protocols.

Routing recovery in ARF is simply based on the control of the transmission interval for routing control messages. In a conventional routing protocol, the transmission interval is fixed at very large value to reduce the energy consumption due to message transmission. However, a conventional routing protocol with this long fixed interval cannot recover a routing tree destroyed by large-scale failure because the notification of the routing tree’s destruction is spread very slowly. Moreover, even when notification of the routing tree destruction arrives at a sensor node with the proper routing tree, the new routing tree is implemented slowly because long intervals are used to transfer routing control messages with the proper tree information. Therefore, for fast routing recovery, routing control messages with the destruction information and proper routing tree should be transferred quickly in the case of failures.

**Figure 3.**
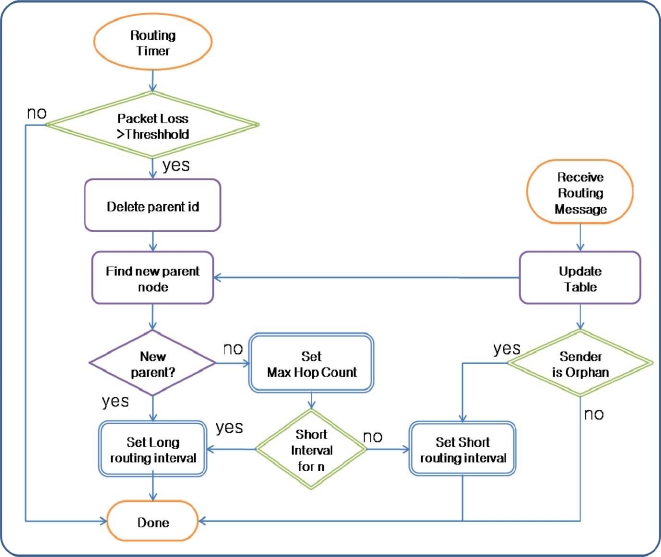
Routing Recovery in ARF.

The transmission interval for routing control messages is controlled adaptively based on the routing state by the Route Recoverer in ARF. The transmission interval for routing control messages is given a long value to reduce the energy consumption under normal circumstances. If the Parent Selector cannot find a parent node in the routing table and the sensor node becomes an orphan node, the Parent Selector starts the Routing Recoverer. The Routing Recoverer sets up a short transmission interval, allowing the routing control message with the Orphan Flag to be transmitted very frequently. Therefore, the failure notification spreads to neighbor nodes quickly. However, to reduce the energy consumption, this short interval is used for a predefined count. In addition, if a sensor node with a correct parent node in the proper routing tree receives a routing control message with an orphan flag, the Routing Recoverer in the sensor node shortens the transmission interval. Therefore, the destroyed routing tree is recovered quickly. Figure 3 shows the routing recovery in ARF.

**Figure 4.**
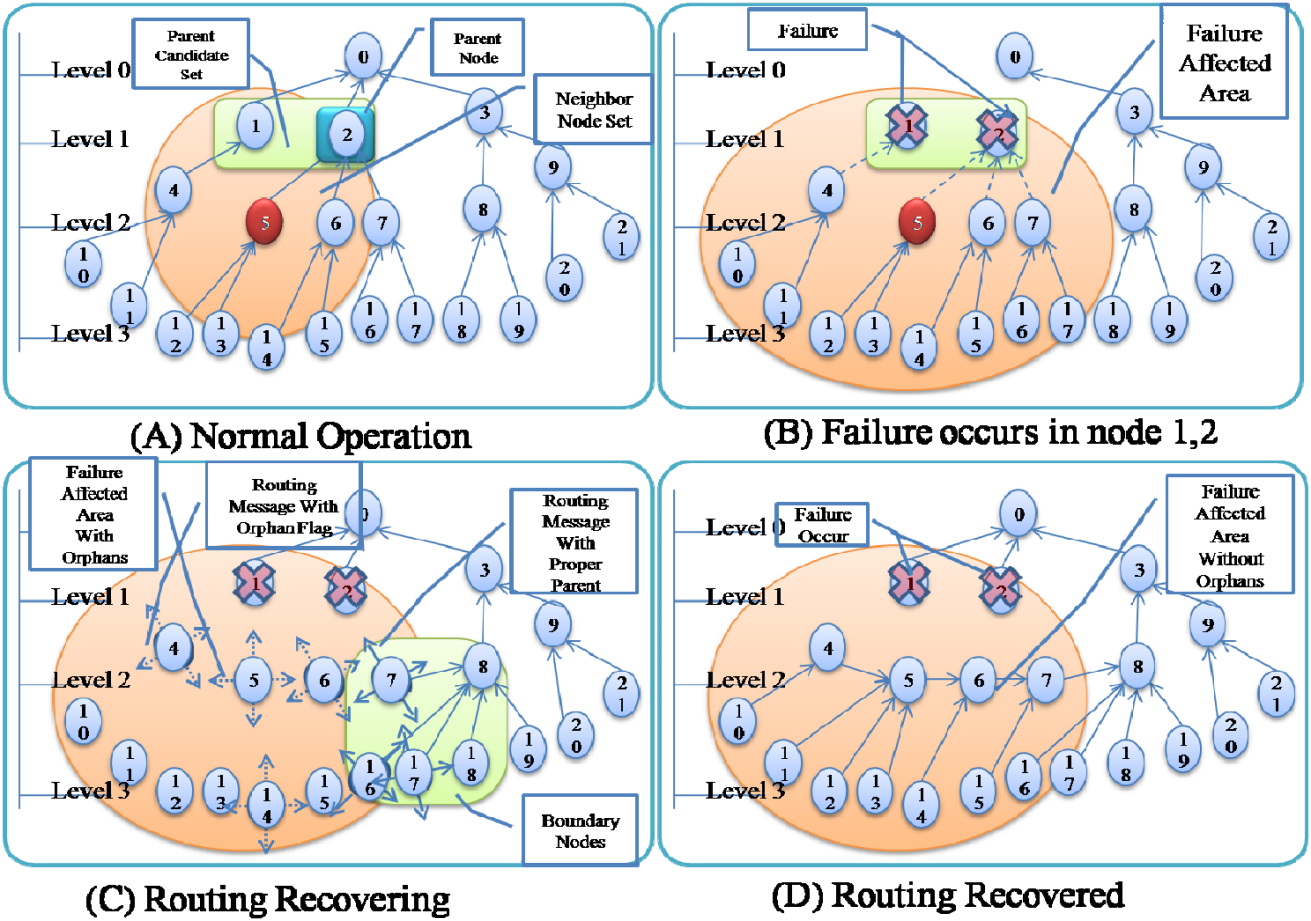
Example of routing recovery.

Figure 4 shows an example of failure occurrence and recovery. Node 0 is a sink node and the levels of the other nodes are based on the hop count from the sink node: nodes 1–3 are in level 1, nodes 4–9 are in level 2, and nodes 10–21 are in level 3. For example, node 5 has two nodes in the upper level (level 1)–nodes 1 and 2–as a parent candidate set and selects node 2 as a parent node (Figure 4A). If nodes 1 and 2 in the upper level fail simultaneously, node 5 cannot find a parent node and sets itself as an orphan node (Figure 4B). Other child nodes of nodes 1 and 2 (nodes 4, 6, 7) in the routing tree also become orphan nodes, and thus routing control messages with orphan flags from these child nodes are transmitted quickly. The child nodes of orphan nodes (nodes 10–15) also become an orphan node and thus transmit a routing control message with orphan flag. If a routing control message with an orphan flag is received by a sensor node with a proper parent node at the boundary of the area affected by the failure, the sensor node shortens its transmission interval (Figure 4C). As routing control messages with proper routing tree information are propagated through the failure affected area, orphan nodes are able to recover the routing tree (Figure 4D).

**Figure 5.**
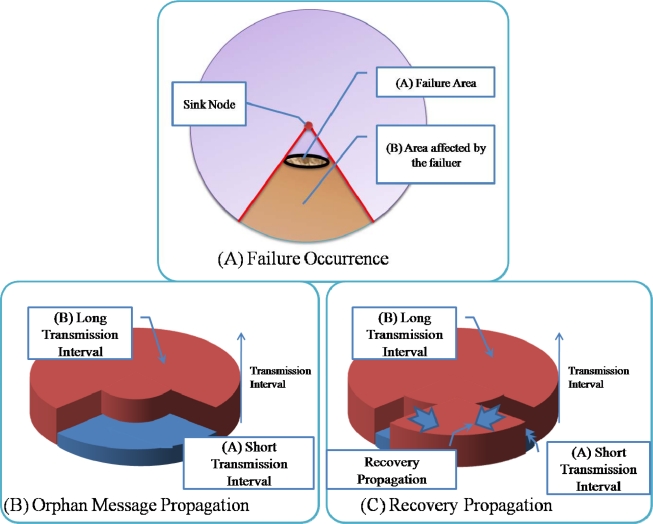
Effect of failure and variation of transmission interval.

As shown in Figure 4, even if only a few nodes (just 2 nodes in Figure 4) fail, numerous child nodes are affected. Sensor nodes at a higher level of the routing tree affect a larger area when they fail (as shown in Figure 5A). In this case, even if routing control messages are transferred quickly from the area with the destroyed routing tree during routing recovery, the routing recovery speed would not be very great. Moreover, the increased number of routing control messages might slow down the recovery speed due to routing control message collisions. To reduce the overhead, ARF restricts the transmission of routing control messages with orphan flags by using short intervals with a predefined count. Therefore, the transmission interval in an area with a destroyed routing tree is set to a short value only while routing control messages with orphan flags are propagated (as shown in Figure 5B). If routing control messages arrive at the boundary area, sensor nodes with proper parents begin to transmit routing control messages with short intervals, and do so until they stop receiving routing packets with orphan flags. Given routing control messages with proper parent nodes, orphan nodes near the boundary could recover from the destroyed routing tree. The boundary moves further toward the center of the affected area, and therefore, the routing tree is reconstructed from the boundary area to the center of the failure affected area (Figure 5C).

The running time of our ARF algorithm is influenced by finding Parent node and implementation of the routing tree. The initialization of fields takes O (V) time where V is the number of average number of neighbor sensor nodes of a sensor node. The operation of computing the link quality takes O (lgV) time. The weight assignment operation takes O (1) time and in fully connected network a node has at most |V − 1| edges. The finding parent candidate Set operation in get Parent procedure takes O (lgV) time. As a node can have maximum V − 1 edges, the loop in get parent runs V − 1 times in a worst case. So the worst case running time of get Parent procedure is O (V lgV). The loop in Routing Tree Reconstruction is executed |V | times and these make a total of O (V 2lgV) running our ARF algorithm.

### 3.3. Fast Initialization

**Figure 6.**
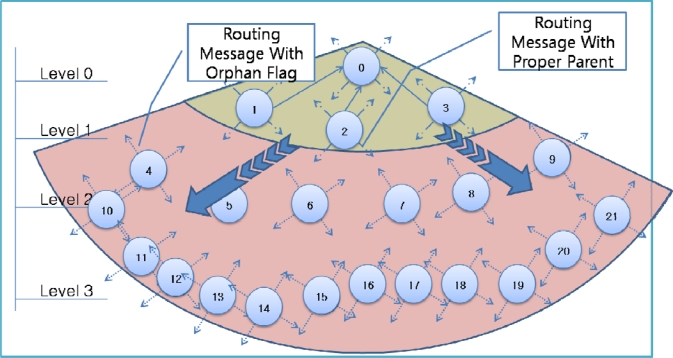
Construction of routing tree in initial stage.

When sensor nodes are initially deployed, they do not have routing information. As routing control messages from a sink node are propagated through the network, a routing tree is constructed. With conventional routing protocols, it takes a very long time to construct a proper routing tree that includes every sensor node. This time increases in proportion to the size of the sensor network, with more sensor nodes in the network requiring more time for routing tree construction. ARF could reduce the construction time needed for a routing tree. This initial stage without a proper routing tree could be regarded as the failure of the entire sensor network. In this case, only the sink node has the proper routing tree and all of the other nodes are orphans. Therefore, the other nodes shorten their routing control message transmission intervals and propagate routing control messages with orphan flags. Sensor nodes around the sink node can receive routing control messages directly from the sink node and select the sink node as a parent node. After that, these nodes start the Routing Recoverer, which transfers routing control messages with the proper parent, as shown in Figure 6. This operation is spread from the sink node to leaf nodes.

**Figure 7.**
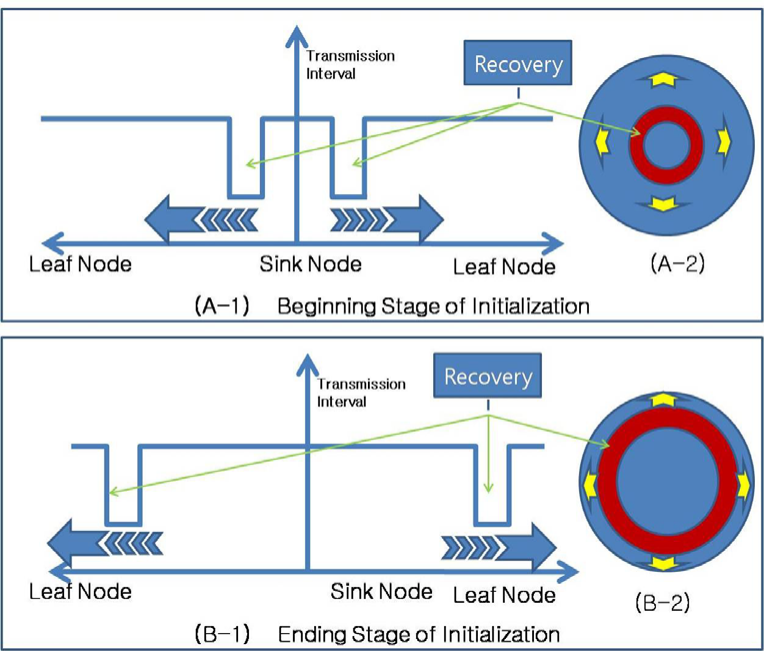
Routing Recovery propagation in initial stage.

Figure 7 shows the propagation of routing recovery at network initialization, with the sink node at the center of the circle shaped network. At the beginning stage of initialization, sensor nodes near the sink node become boundary nodes and shorten their transmission intervals, while sensor nodes far from the sink node set long transmission intervals. As the routing tree is constructed, the boundary moves toward the outside of the network, and therefore, the routing recovery process using the short intervals also moves toward the outside.

For the routing control message transmissions from a sensor node to neighbor sensor nodes, CSMA is used to reserve the channel. We assume that each node knows the number of contending neighbor nodes (m) and contends the channel with the optimal probability p = 1/m. The probability that one contending node wins the channel is p_succ_ = (1 − 1/m)^m−1^. Since the number of slots needed until the successful reservation is a geometric random variable, the average number of contending slots (ACS) is given by:

(1) $$\mathrm{ACS}=\frac{1}{{\left(1-\frac{1}{M}\right)}^{M-1}}$$

where M is the Average number of Neighbor nodes.

The transmission interval of routing control message is T, the average transmission time of routing message in single hop (ATS) is T + ACS and the total propagation time of routing control message from a sink node to a leaf sensor node in level L (TPT) is:

$$\mathrm{TPT}=L\times \mathrm{ATS}=L\times (T+\mathrm{ACS})=L\times \left(T+\frac{1}{{\left(1-\frac{1}{M}\right)}^{M-1}}\right)$$

The initialization time of a routing tree is in proportion to the number of level L and the routing interval T and thus decreasing the routing interval T can decrease the initialization time of routing tree when the number of level L is fixed.

## 4. Performance

### 4.1. Experimental Setup

To evaluate the performance of the proposed ARF, we simulated MintRoute, Proc, and ARF together using TOSSIM, which is a simulator for TinyOS [26–27]. TinyOS is an operating system developed for event based sensor networks at UC Berkeley. TOSSIM provides a simulation environment that simulates a real sensor network with TinyOS. An application in TinyOS consists of components from each network layer and hardware. The application running on TOSSIM could be run on real sensor nodes such as a micaz or a telos. Thus, we could say that our implemented simulation reflects the real world. Table 3 lists the specifications for the simulated sensor mote.

**Figure 8.**
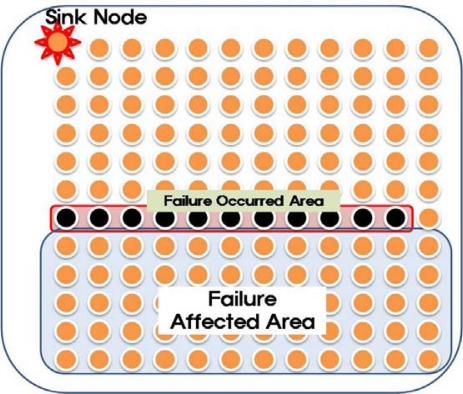
Sensor node placement in simulation.

In the simulation, 144 sensor nodes sent data to a sink node every second, as shown in Figure 8. The total simulation time was 600 seconds and at 200 seconds form the simulation start, some sensor nodes in the middle area (Failure Occurred Area in Figure 8) failed concurrently, leaving only one sensor node as a path through the failure area. We selected this setting to show the worst case of failures in the network. In this case, most of nodes in failure affected area lost the parent and had to reselect the parent node. Moreover, there is only a node which has a proper parent and thus the routing recovery has to start from this node.

Within TinyOS, MintRoute is the standard routing protocol. MintRoute is a proactive routing protocol, in which sensor nodes send routing control messages periodically with notifications of their local states. Although MintRoute detects the failure of a neighbor node with packet loss, just like ARF, MintRoute tries to select a parent node at the same level. The Proc protocol detects the failure of a parent node with Ack packets and changes the parent node whenever a sensor node cannot receive an Ack packet from its parent node. Both MintRoute and Proc use a fixed transmission interval for routing control messages, while ARF changes the interval adaptively based on the state of the network. In the simulations, the intervals for both MintRoute and Proc were set to 5,000 ms to simulate a short interval and 10,000 ms to simulate a long interval. ARF used intervals of 5,000 ms in the case of failure detection and 20,000 ms in the case of a normal state. We evaluated the number of packets received by a sink node, the number of packets broadcast for routing, and the energy consumption. In the graph, RPOC-5000 indicates the Proc protocol with the 5,000 ms interval, PROC-10000 indicates the Proc protocol with the 10,000 ms interval, MINT-5000 indicates the MintRoute with 5,000 ms, and MINT-10000 indicates the MintRoute with 10,000 ms, respectively

### 4.2. Initial Stage

**Figure 9.**
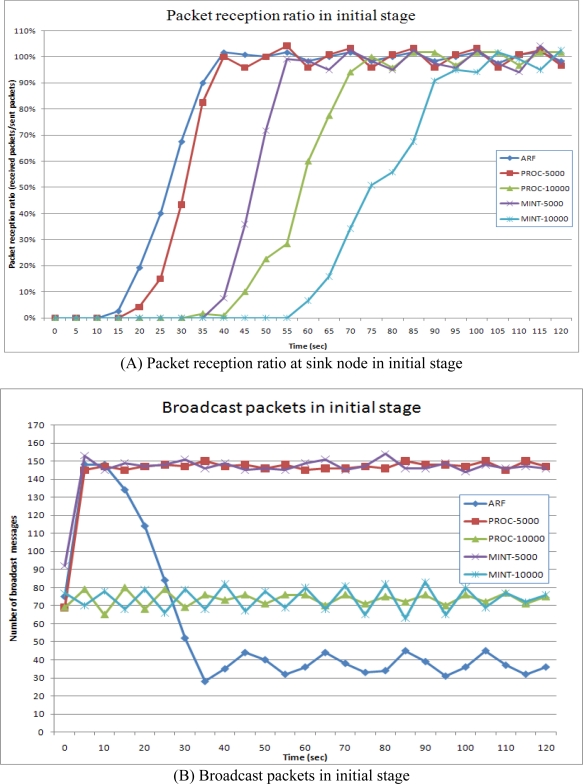
Packet reception ratio and broadcast packets in initial stage.

The sensor nodes tried to send data packets to the sink node when there was a path to it. Figure 9A shows the packet reception ratio at the sink node in the initial stage. The successfully received packets sent from 72 sensor nodes in the failure affected area were counted every second and divided by the number of sent packets. Figure 9B shows the number of broadcast packets sent by the 72 sensor nodes. The broadcast packets were used for routing control messages.

In Figure 9A, the 100-s period from 0 s to 100 s shows the initial stage of network configuration. In this period, ARF showed the fastest performance in the construction of a routing tree. Proc and MintRoute with 5,000 ms intervals showed similar performances. As explained in Section 3.3, the initial stage could be regarded as the failure of the entire network, and thus, routing protocols with short transmission intervals could be used to construct the routing tree. ARF set the routing interval at 5,000 ms for failure and routing recovery and thus showed a fast routing construction speed. In the initial stage, all of the sensor nodes in ARF broadcast routing control messages with orphan flags in failure detection for a while, and the sensor nodes near the sink node started to broadcast routing control messages with the proper routing tree. As the routing tree was being constructed from the sink node to the leaf nodes, the sensor nodes with a parent node increased their routing interval from 5,000 ms to 20,000 ms. As a consequence, the number of broadcasts in ARF increased drastically between 0 s to 10 s and then decreased slowly between 20 s and 35 s, as shown in Figure 9B. The number of broadcast messages in Proc and MintRoute shows almost constant since Proc and MintRoute use fixed routing interval. The Proc protocol with 5,000 ms also constructed the routing tree quickly, similar to ARF, but the number of broadcast packets remained at a high rate. MintRoute with the 5,000 ms interval also showed a high rate of message routing, while the packet reception ratio was very low compared to ARF and Proc with 5,000 ms. Both Proc and MintRoute with the 10,000 ms interval transferred fewer routing control messages but the routing tree was constructed very slowly. As a result, ARF could construct fast the routing tree with fewer routing control messages at the initial stage.

### 4.3. Routing Recovery

**Figure 10.**
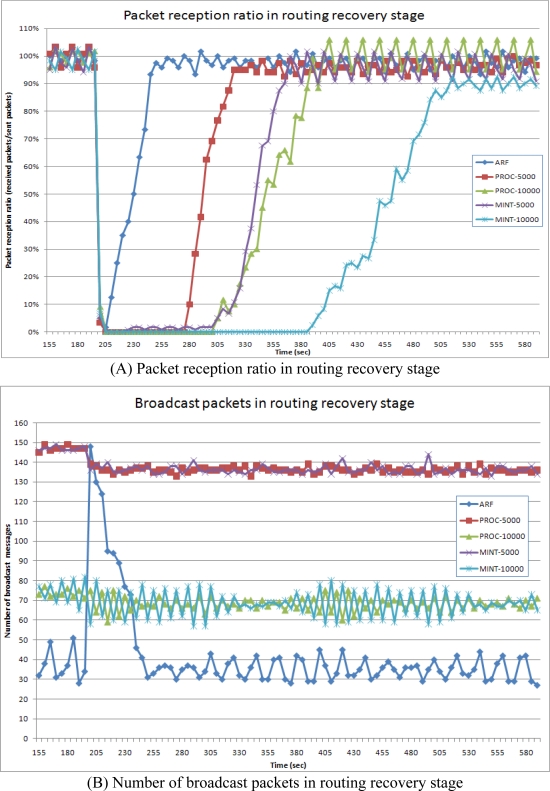
Packet reception ratio and number of packets in routing recovery stage.

Figure 10 shows the packet reception ratio and the number of broadcast packets during the routing recovery stage. The failures occurred at 200 s when 11 sensor nodes were simultaneously turned off. Out of the 144 sensor nodes, 72 were affected by the failure.

ARF showed the fastest routing recovery, requiring only 50 s, from 200 s to 250 s. ARF began the routing recovery when the failure was detected. Thus, packets were received immediately. ARF only selects a parent from the highest level of the routing tree and thus could detect the failure quickly. Moreover, the sensor nodes in the boundary area transmitted routing control messages at short intervals, allowing the sensor nodes near the boundary area to recover the routing tree quickly. As a result, the number of packets broadcast by ARF increased drastically at 200 s, but decreased again as the routing tree was being recovered between 200 s and 250 s. After 250 s, the entire routing tree was properly reconstructed, all of the data packets were received by sink node, and the number of broadcast packets decreased to the lowest level because the routing control messages were transmitted at 20,000 ms intervals. Proc with the 5,000 ms interval showed the next best routing recovery speed, maintaining a high rate of broadcast packets. However, it took packets 80 s to arrive at the sink node. During these 80 s, sensor nodes in the failure affected area were busy finding a new parent from all of their neighbor nodes. Proc with the 10,000 ms interval showed low rate of broadcast packets but the routing tree was recovered at a lower speed. MintRoute showed a very slow routing recovery speed for both the 5,000 ms and 10,000 ms intervals. As a result, ARF could recover the routing tree fast with fewer routing control messages in case of large area failure.

### 4.4. Total Packets

**Figure 11.**
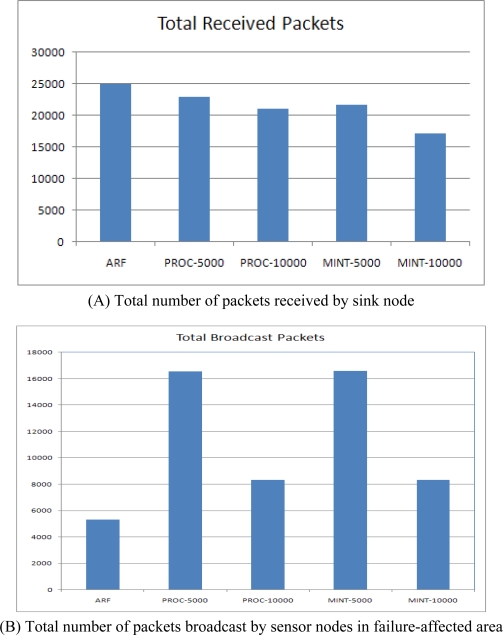
Total number of received packets and broadcast packets.

Figure 11 shows the total number of packets received by the sink node and the total number of broadcast packets sent by sensor nodes in the failure affected area. ARF showed the highest packet reception with the lowest broadcast packets. ARF recovered the destroyed routing tree as soon as the failure occurred and thus most of the packets were received, showing the highest packet reception. At the initial stage, a short transmission interval for routing control messages was only used for 20 s and for 50 s for routing recovery, while it was set at the longer interval during other periods. Therefore, the number of broadcast packets was very low. In contrast, both Proc and MintRoute with 5,000 ms (short interval) showed high packet reception levels but the number of broadcast packets was also very high. Both Proc and MintRoute with 10,000 ms (long interval) showed low packet reception levels with a small number of broadcast packets. As a consequence, ARF can collect more data with less message broadcast overhead than other protocols.

### 4.5. Energy Consumption

**Figure 12.**
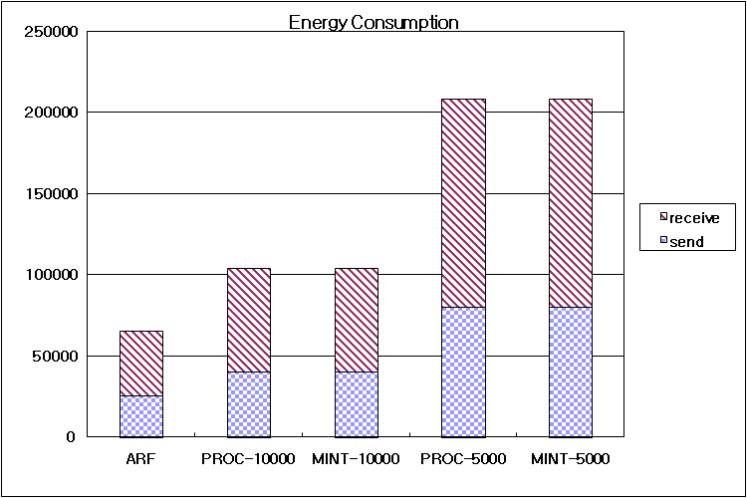
Energy consumption.

Figure 12 shows the energy consumed by the routing protocols. The energy was computed by counting the number of transmitted broadcast and the number of received broadcast packets. ARF showed the lowest energy consumption because it broadcast the routing control messages at short intervals only during routing recovery, while it used long intervals in other cases. Therefore, the total number of sent and received routing packets was very low. Both Proc and MintRoute with the long interval (5,000 ms) showed low energy consumptions with low numbers of broadcast packets, but the received packet totals were also low. Proc and MintRoute with the short interval (10,000 ms) showed the highest energy consumption levels because these protocols always transmitted routing control messages at short intervals. As a result, ARF could recover a destroyed routing tree due to a large failure at a fast speed while consuming less energy.

## 5. Conclusions

Large-scale wireless sensor networks are expected to play an increasingly important role in data collection in hazardous areas. However, the physical fragility of sensor nodes makes reliable routing in hazardous areas a challenging problem. Because several sensor nodes in a hazardous area could be damaged simultaneously, a network should be able to recover routing after node failures over large areas. Many routing protocols take into account recovery after the failure of a single node, but it is difficult for these protocols to recover the routing after large-scale failures. In this paper, we propose a routing protocol, referred to as ARF, which allows a network to recover quickly from failures over large areas. ARF detects failures by counting the packet losses from a parent node, and upon failure detection, ARF decreases the routing interval to notify the neighbor nodes of the failure. Our experimental results indicate clearly that ARF could recover from large-area failures quickly with less packets than previous protocols.

## Figures and Tables

**Table 1.** ARF components and their functions.

**Component**	**Function**
Routing Table	Lists neighbors, routing information, and link quality
Table Manager	Inserts and evicts neighbors from routing table
Link Estimator	Computes link quality
Parent Selector	Selects a parent from the routing table
Cycle Detector	Detects and removes loops
Forwarding Module	Forwards packets toward the sink node
Routing Recoverer	Recovers a destroyed routing tree
Timer	Periodically broadcasts the routing table and selects a parent
Dispatcher	Dispatches packets from the receive queue

**Table 2.** Routing table of ARF.

Node ID	Hop Count	Parent ID	Transfer Packets	Received Packets		Packet Seq.	Lost Packets	Link Quality	Orphan Flag
				Data	Routing				
									

**Table 3.** Specifications for simulated sensor mote.

MCU	ATMEGA 128L 8MHz
Memory	4K RAM / 128K FLASH
RF Transceiver	Chipcon CC2420 IEEE 802.15.4/ZigBee compliant 2.4 GHz Frequency band 250 Kbps Transmit data rate −24 dBm to 0 dBm RF power 20 m to 30 m indoor Range
